# Supplementary material for: Melting and defect transitions in FeO up to pressures of Earth’s core-mantle boundary
Source: Nat Commun. 2023 Nov 13;14:7336. doi: 10.1038/s41467-023-43154-w (PMC10643405; doi:10.1038/s41467-023-43154-w)
Supplement: Supplementary file 1 — Supplementary Information [file 41467_2023_43154_MOESM1_ESM.pdf]

Supplementary Material for Melting and defect transitions in FeO up to pressures of Earth's core-mantle boundary

Vasilije V. Dobrosavljevic[1,5]*, Dongzhou Zhang[2], Wolfgang Sturhahn[1], Stella Chariton[3], Vitali B. Prakapenka[3], Jiyong Zhao[4], Thomas S. Toellner[4], Olivia S. Pardo[1,6], Jennifer M. Jackson[1]

[1]Seismological Laboratory, Division of Geological and Planetary Sciences, California Institute of Technology; Pasadena, CA, USA.

[2]Hawai‘i Institute of Geophysics and Planetology, University of Hawai‘i at Mānoa; Honolulu, HI, USA.

[3]Center for Advanced Radiation Sources, The University of Chicago; Chicago, IL, USA.

[4]Advanced Photon Source, Argonne National Laboratory; Chicago, IL, USA.

[5]Now at Earth and Planets Laboratory, Carnegie Institution for Science, Washington D.C., USA.

[6]Now at Physics Division, Physical & Life Sciences Directorate, Livermore, CA, USA.

*Corresponding author. Email: vasilije@carnegiescience.edu

Contents:

Figures

S1 – Additional XRD patterns
S2-6 – All XRD heating runs
S7-9 – All SMS heating runs
S10-13 – All SMS time spectra
S14 – Thermal pressure

Tables

S1-3 – Transition *P*-*T* conditions
S4-14 –Fitting results for all SMS time spectra

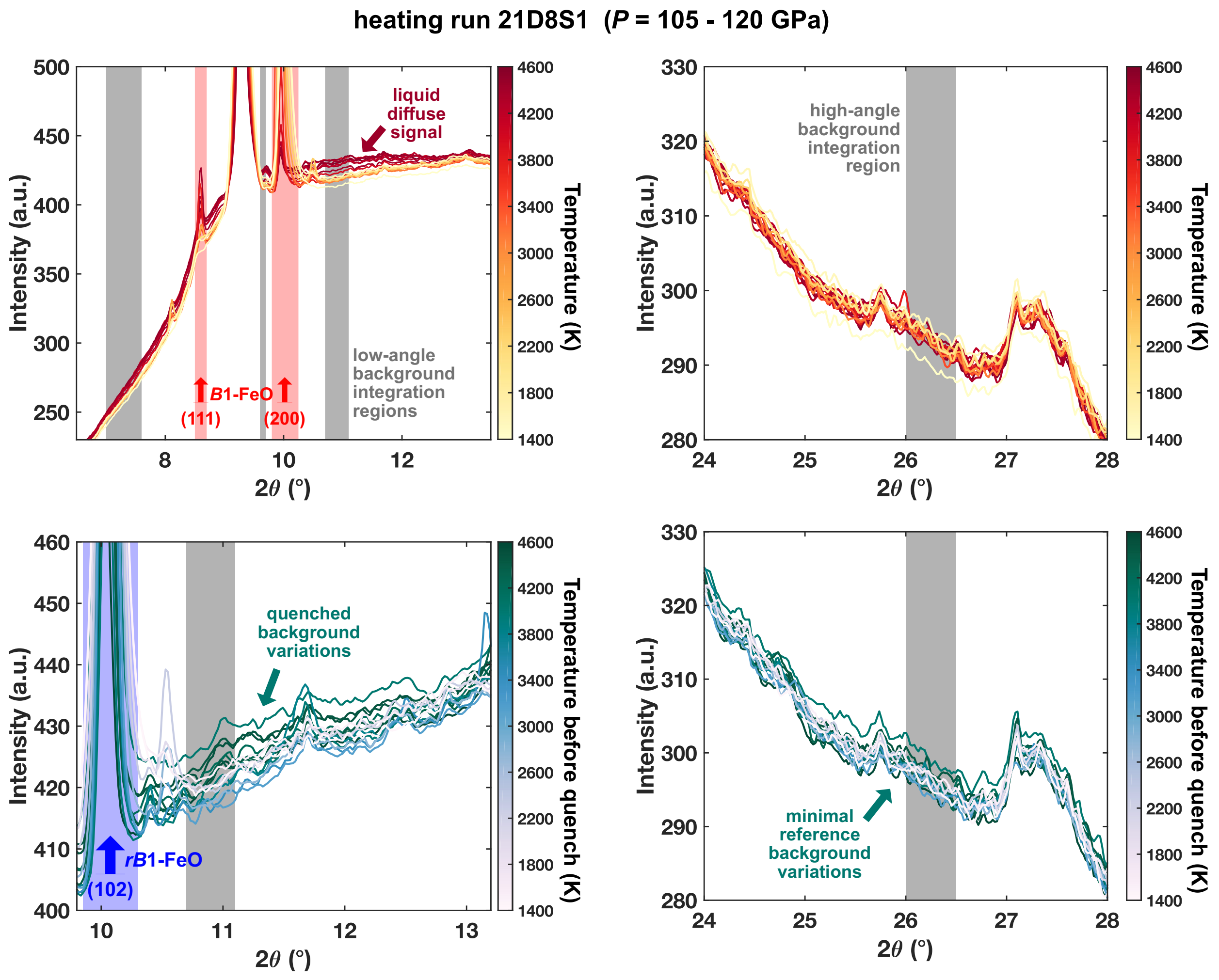

Fig. S1.

XRD heating run 21D8S1. Top panels show high-temperature integrated patterns. Shaded bars indicate FeO reflections (red) and background integration regions (gray). Bottom panels show quenched integrated patterns. Background intensities at low angles relative to high angles vary during the heating run (Fig. 3).

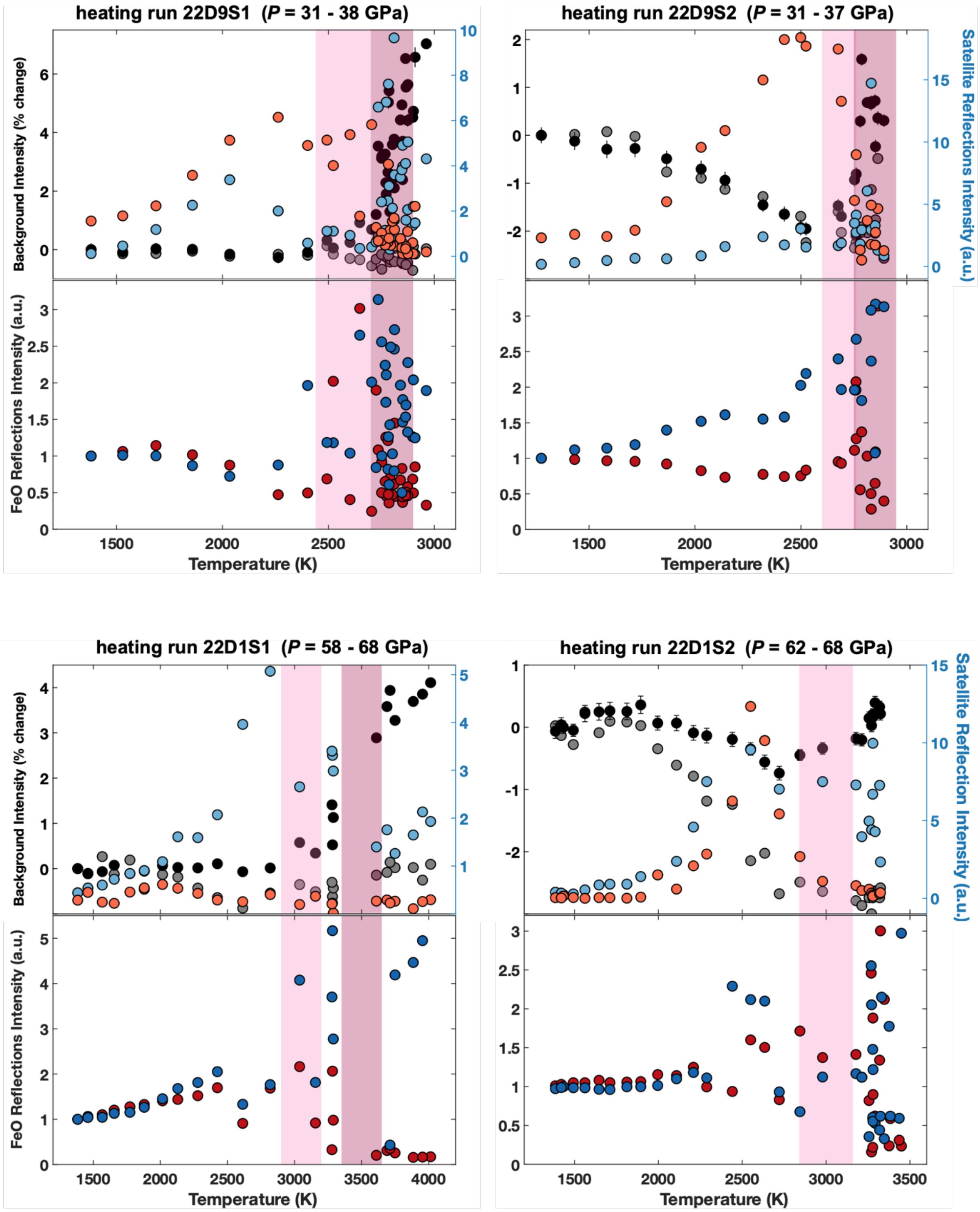

Fig. S2.

XRD heating runs 22D9S1, 22D9S2, 22D1S1, and 22D1S2. Format of panels and meaning of colors for data and shaded boxes are identical to Fig. 2.

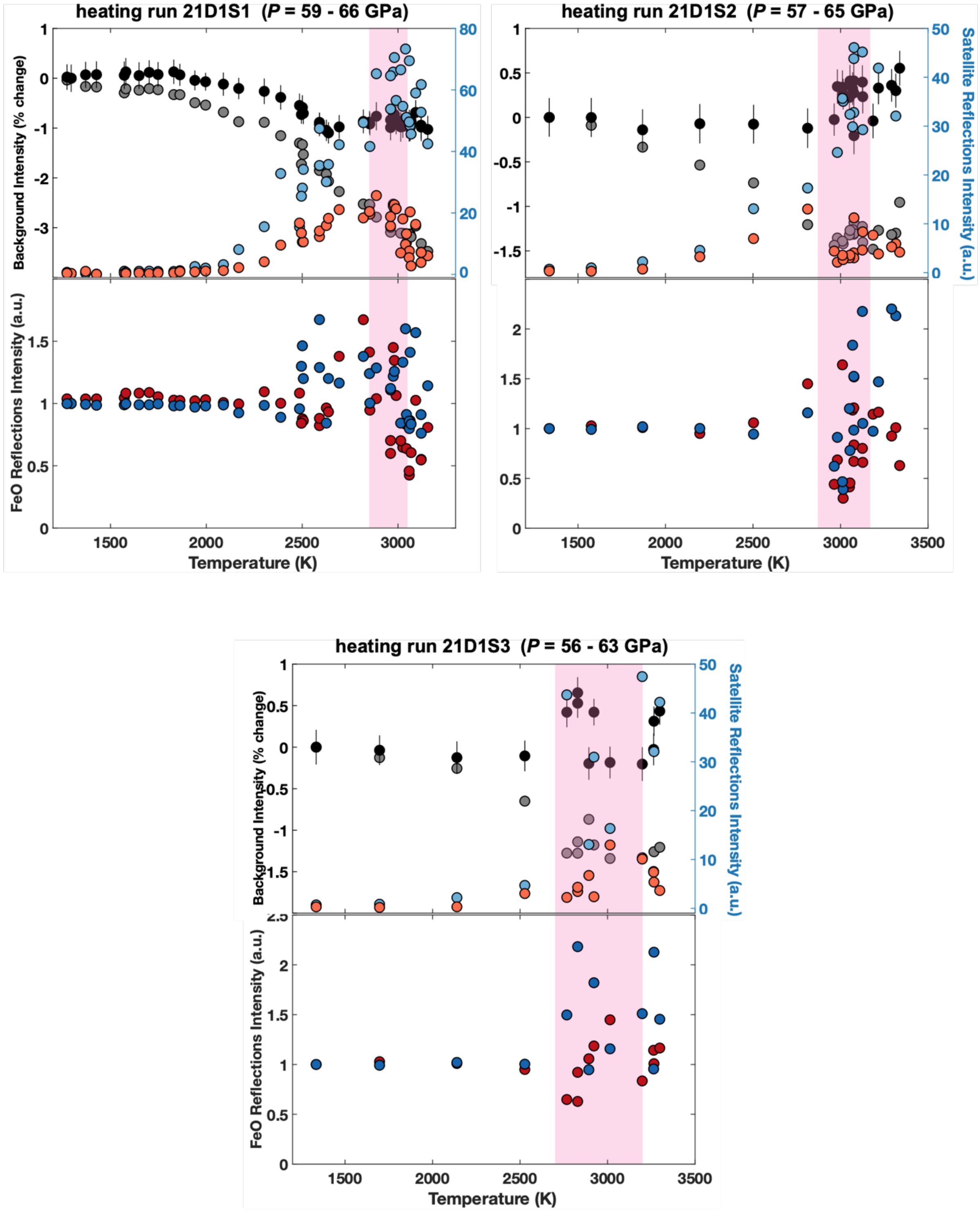

Fig. S3.

XRD heating runs 21D1S1, 21D1S2, and 21D1S3. Format of panels and meaning of colors for data and shaded boxes are identical to Fig. 2.

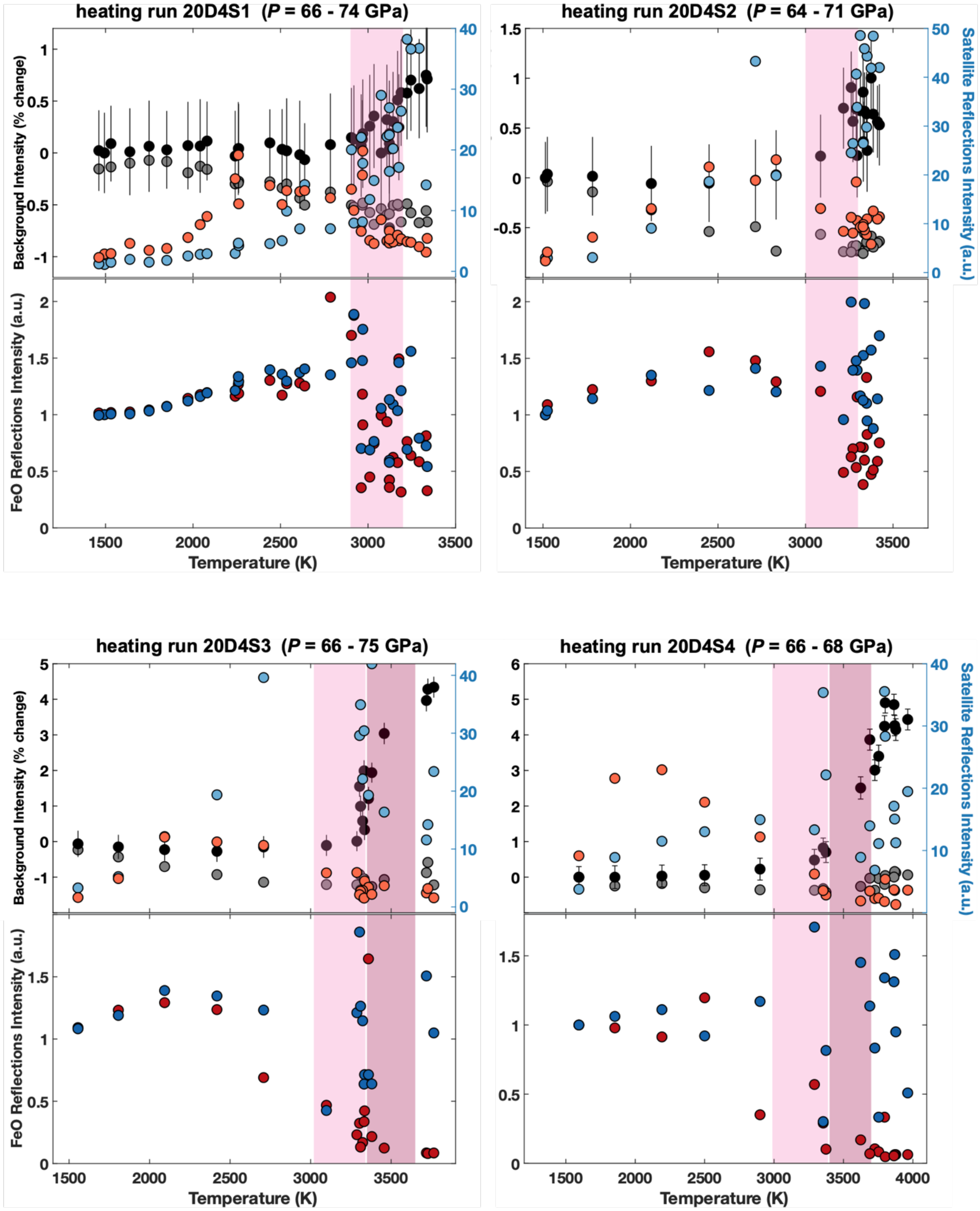

Fig. S4.

XRD heating runs 20D4S1, 20D4S2, 20D4S3, and 20D4S4. Format of panels and meaning of colors for data and shaded boxes are identical to Fig. 2.

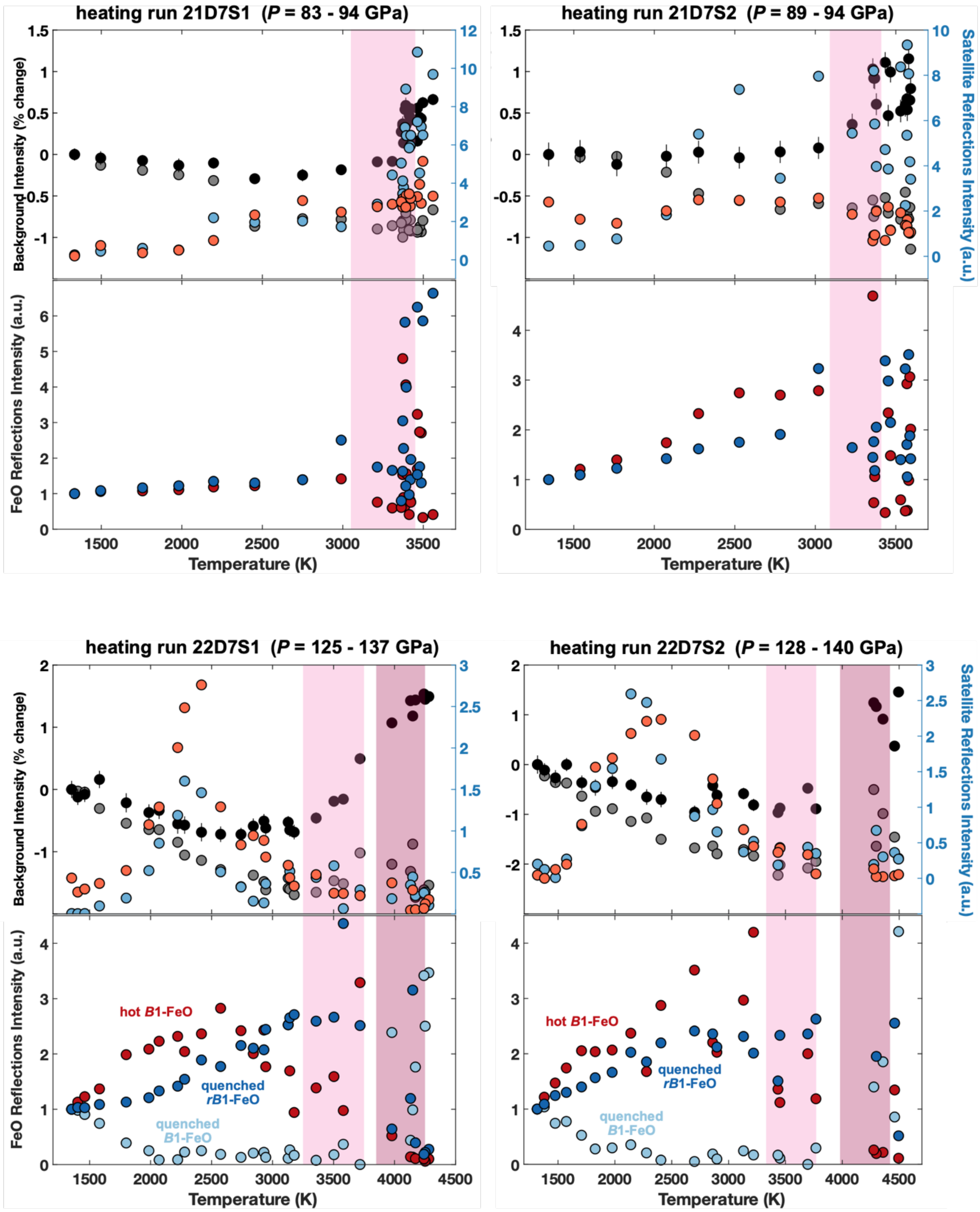

Fig. S5.

XRD heating runs 21D7S1, 21D7S2, 22D7S1, and 22D7S2. Format of panels and meaning of colors for data and shaded boxes are identical to Fig. 2.

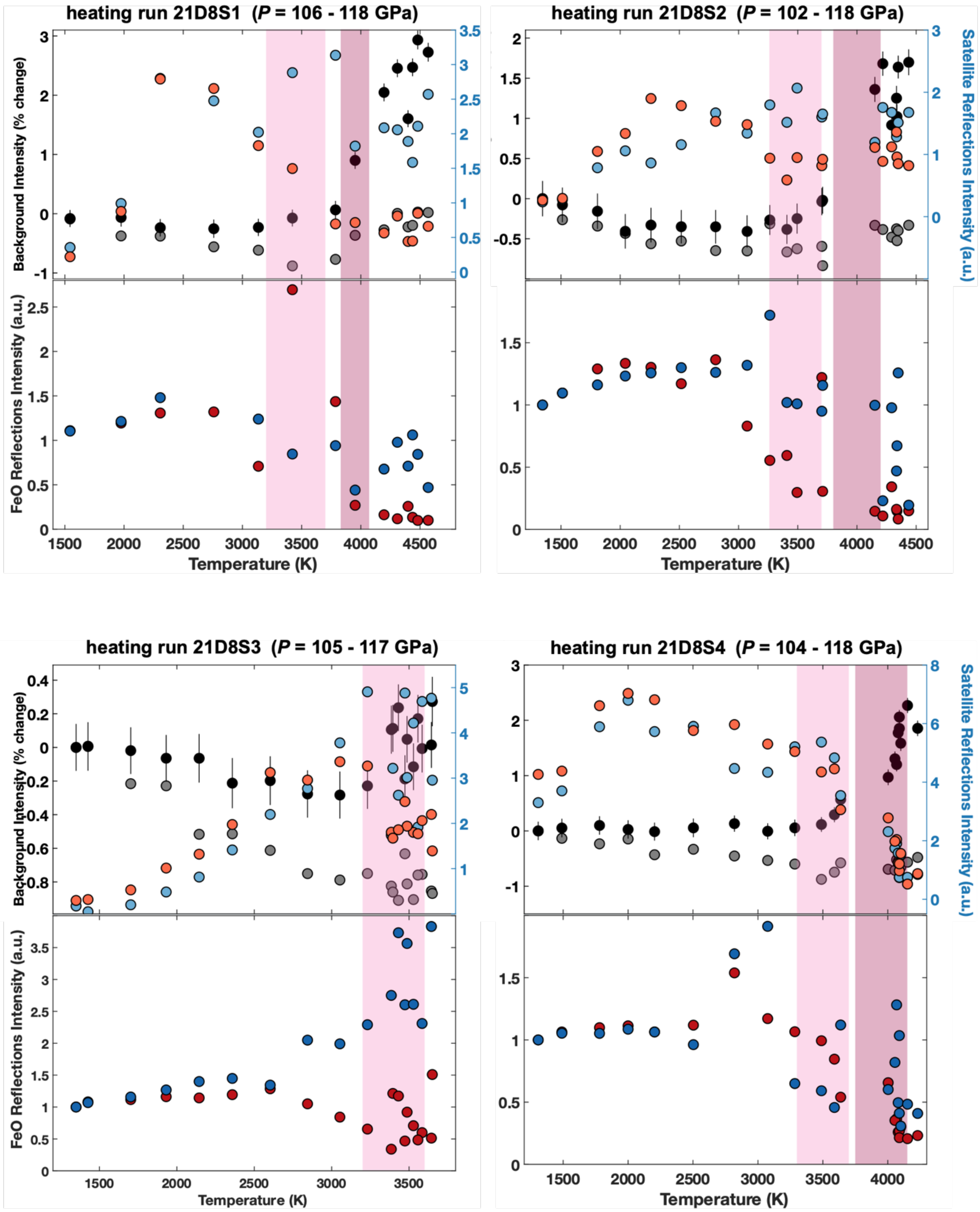

Fig. S6.

XRD heating runs 21D8S1, 21D8S2, 21D8S3, and 21D8S4. Format of panels and meaning of colors for data and shaded boxes are identical to Fig. 2.

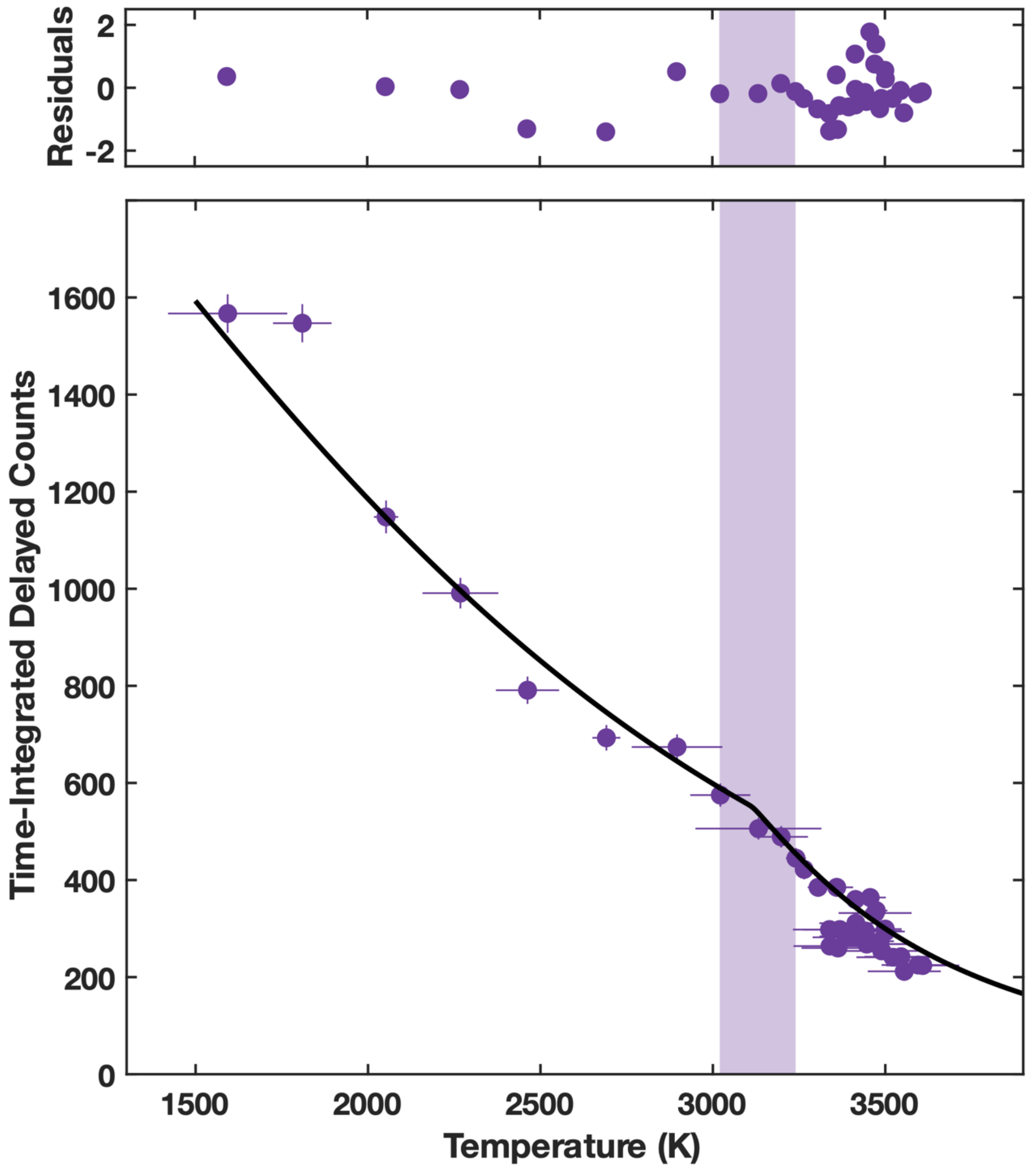

Fig. S7.
SMS heating run 18D4P3 (P_{melt} = 48 GPa). Format of panels and meaning of colors for data, curve, and shaded boxes are identical to Fig. 3.

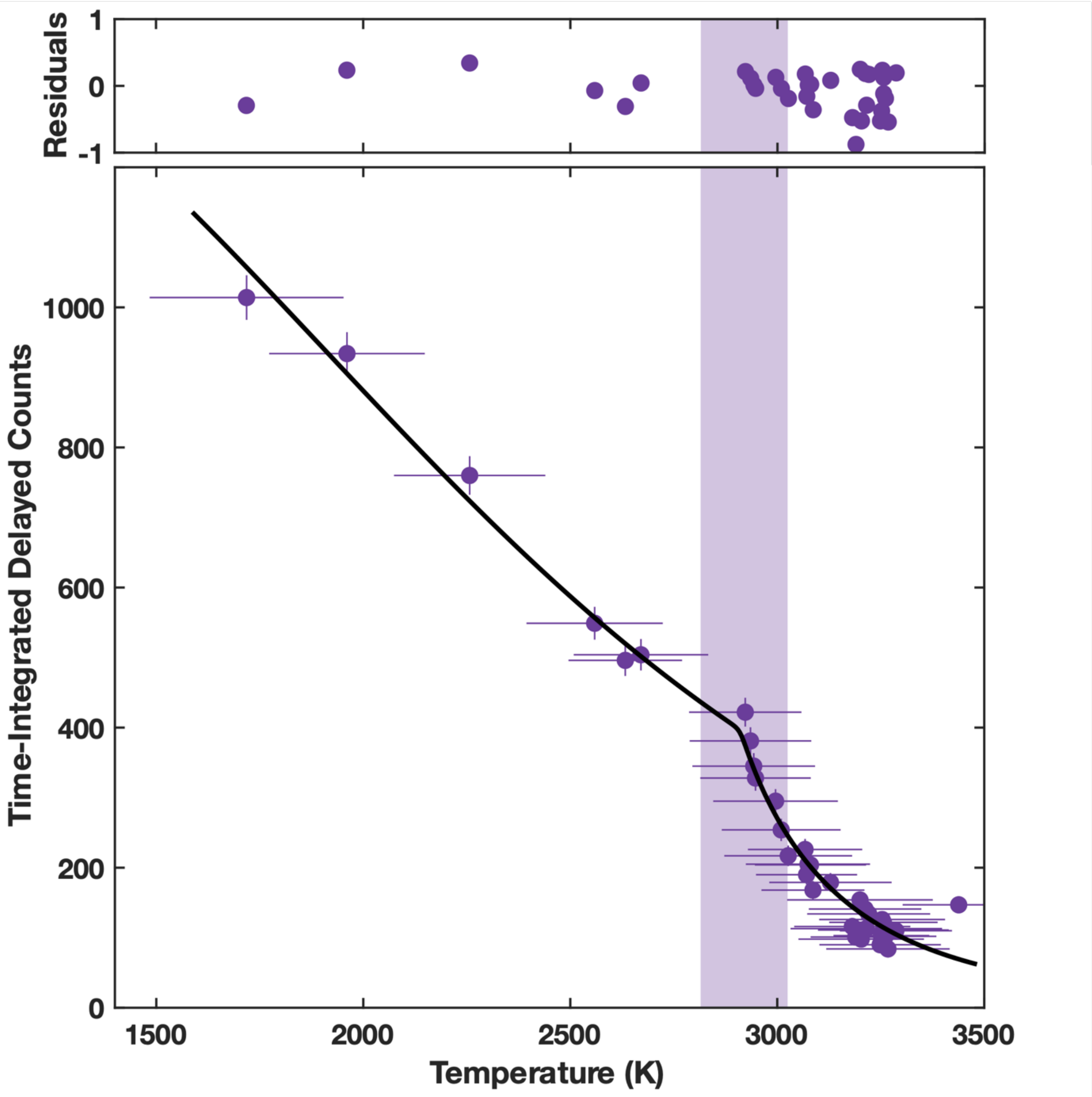

Fig. S8.
SMS heating run 18D4P2 (P_{melt} = 37 GPa). Format of panels and meaning of colors for data, curve, and shaded boxes are identical to Fig. 3.

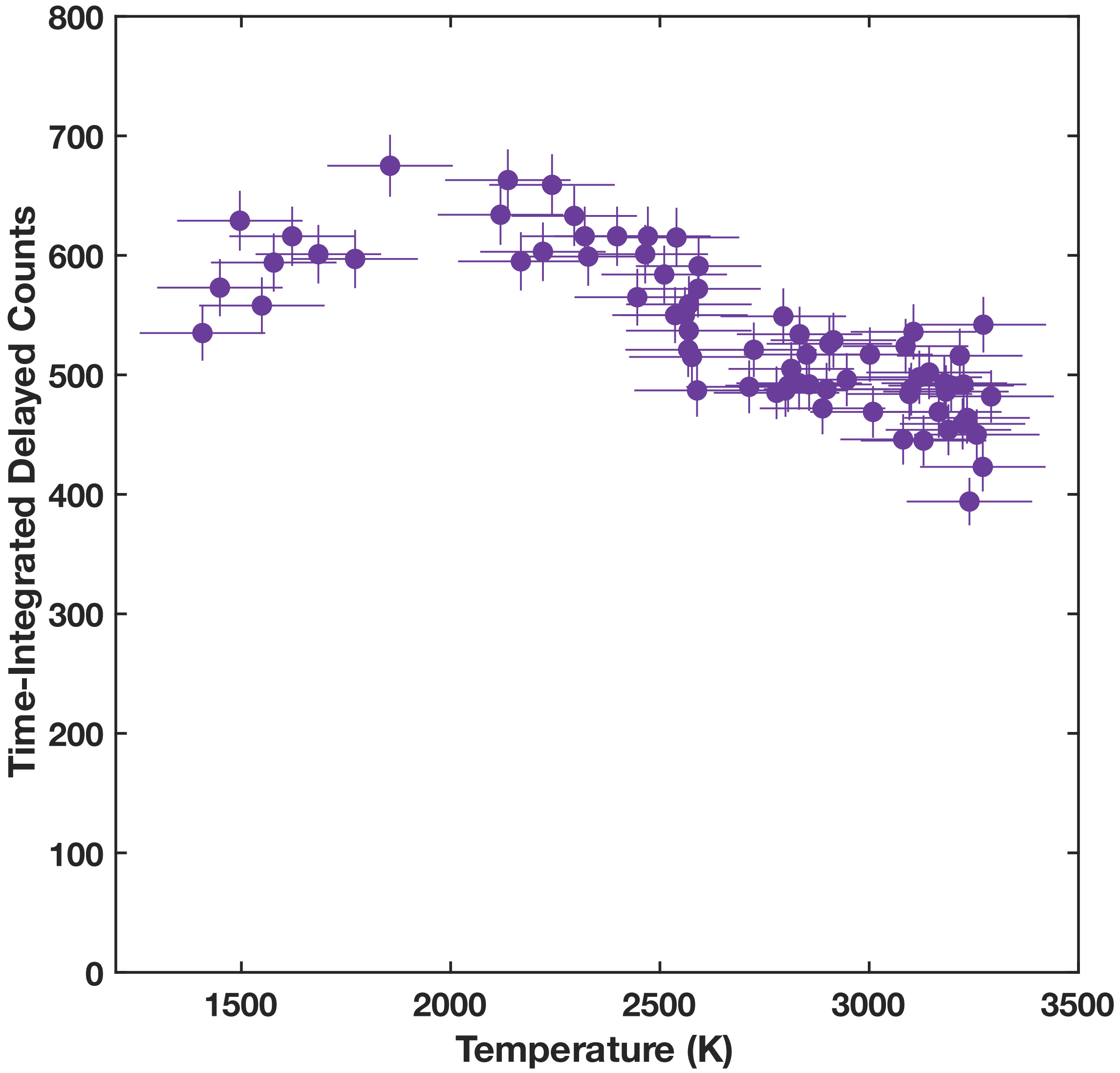

Fig. S9.

SMS heating run 19D3p5c (P_{max} = 96 GPa). Format of panels and meaning of colors for data, curve, and shaded boxes are identical to Fig. 3. In this heating run, no signature of melting (loss of Mössbauer signal intensity) was observed up to the highest temperature.

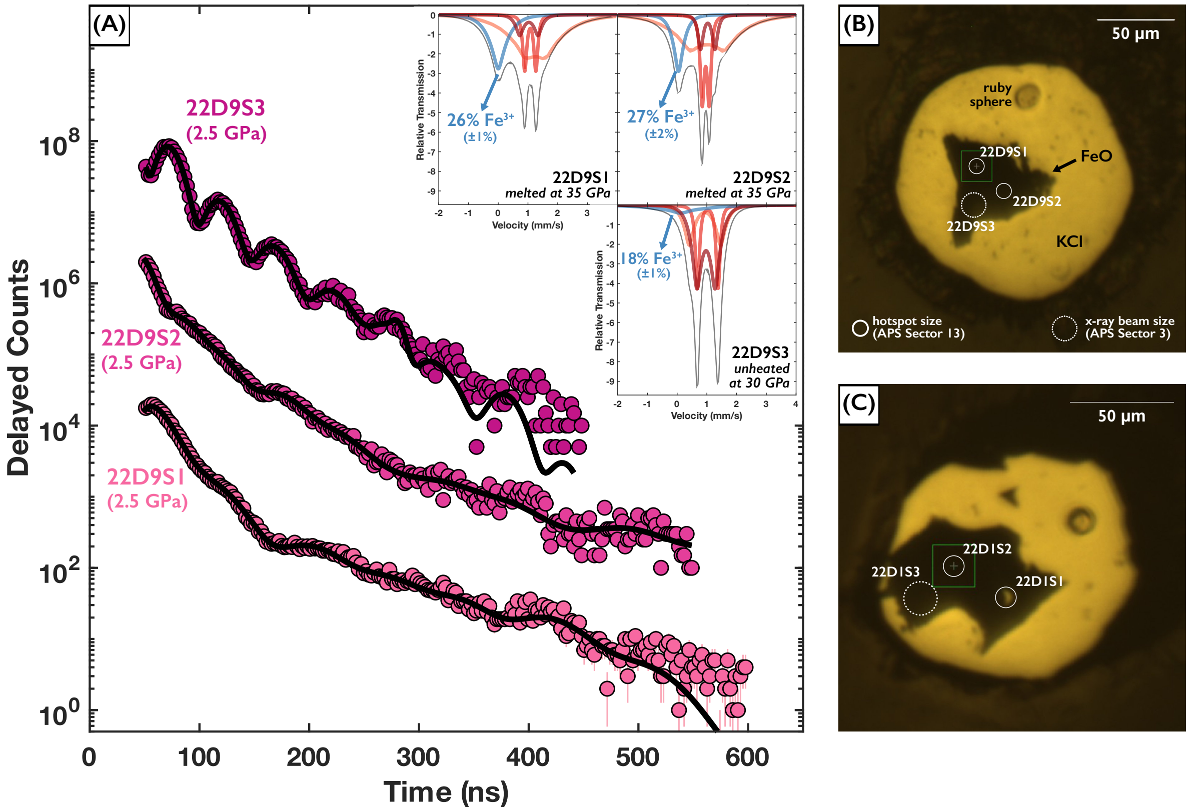

Fig. S10.
(A) SMS time spectra 22D9S1, 22D9S2, and 22D9S3 showing data (colored points) and best-fitting models (black lines). Insets show best-fitting models calculated in energy domain, showing the ferric iron lines in light blue. See Tables S8-10 for full details. (B) and (C) show FeO samples heated at Sector 13 (XRD) and decompressed to ~2.5 GPa for SMS measurements at Sector 3. Circles show locations of heating spots and SMS measurements, with circle sizes representing sizes of the Sector 3 x-ray beam (dashed) and Sector 13 laser hotspot (solid).

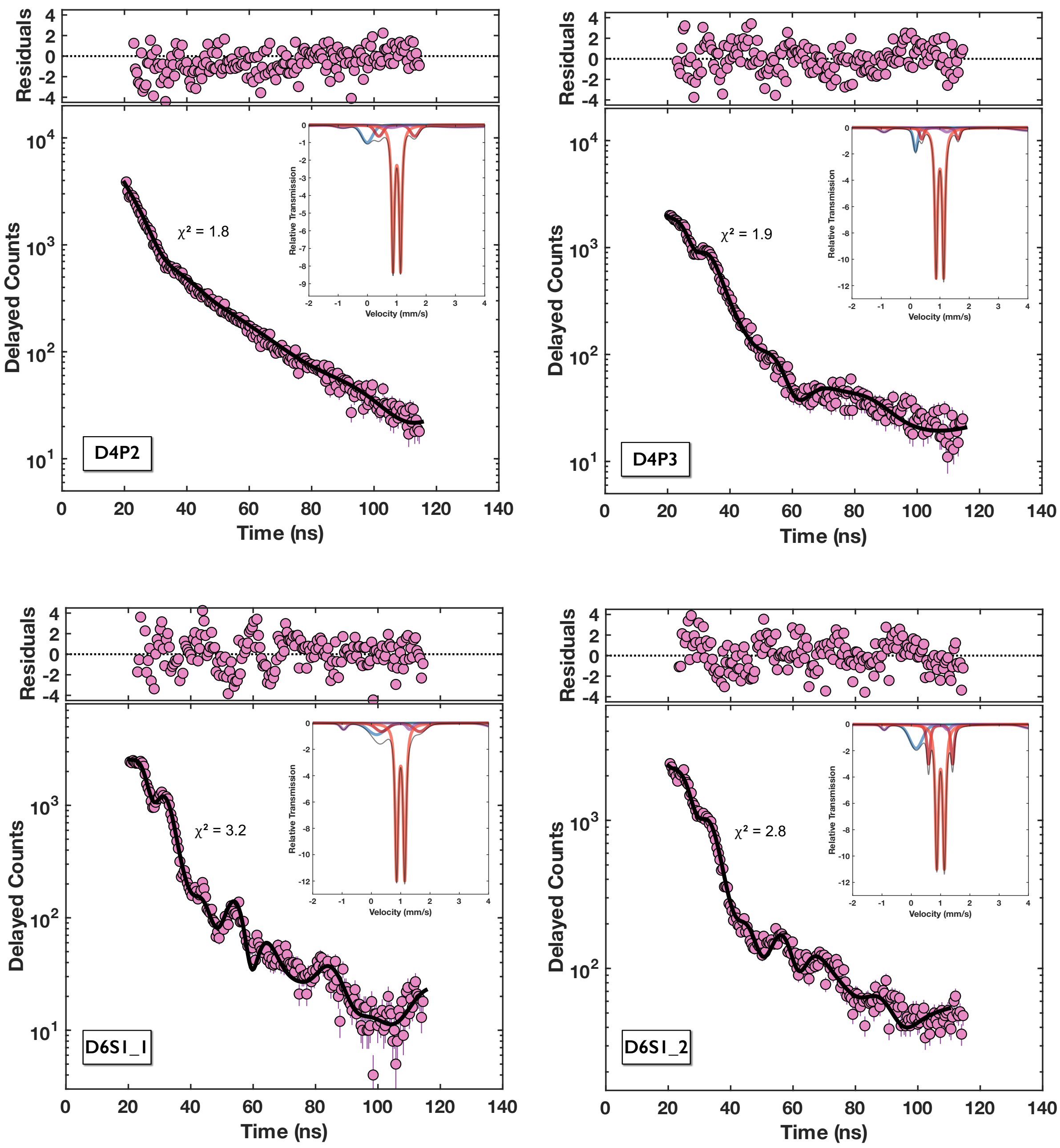

Fig. S11.
SMS time spectra (24-bunch top-up mode) D4P2 (top left), D4P3 (top right), D6S1_1 (bottom left), and D6S1_2 (bottom right) showing data (colored points) and best-fitting models (black lines). D4P2, D4P3, and D6S1_1 are collected before the corresponding heating runs at the annealing temperature of ~1500 K (Tables S4-6), while D6S1_2 is collected after heating run 18D6S1 (Table S7). Insets show best-fitting models calculated in energy domain, showing the ferric iron lines in light blue.

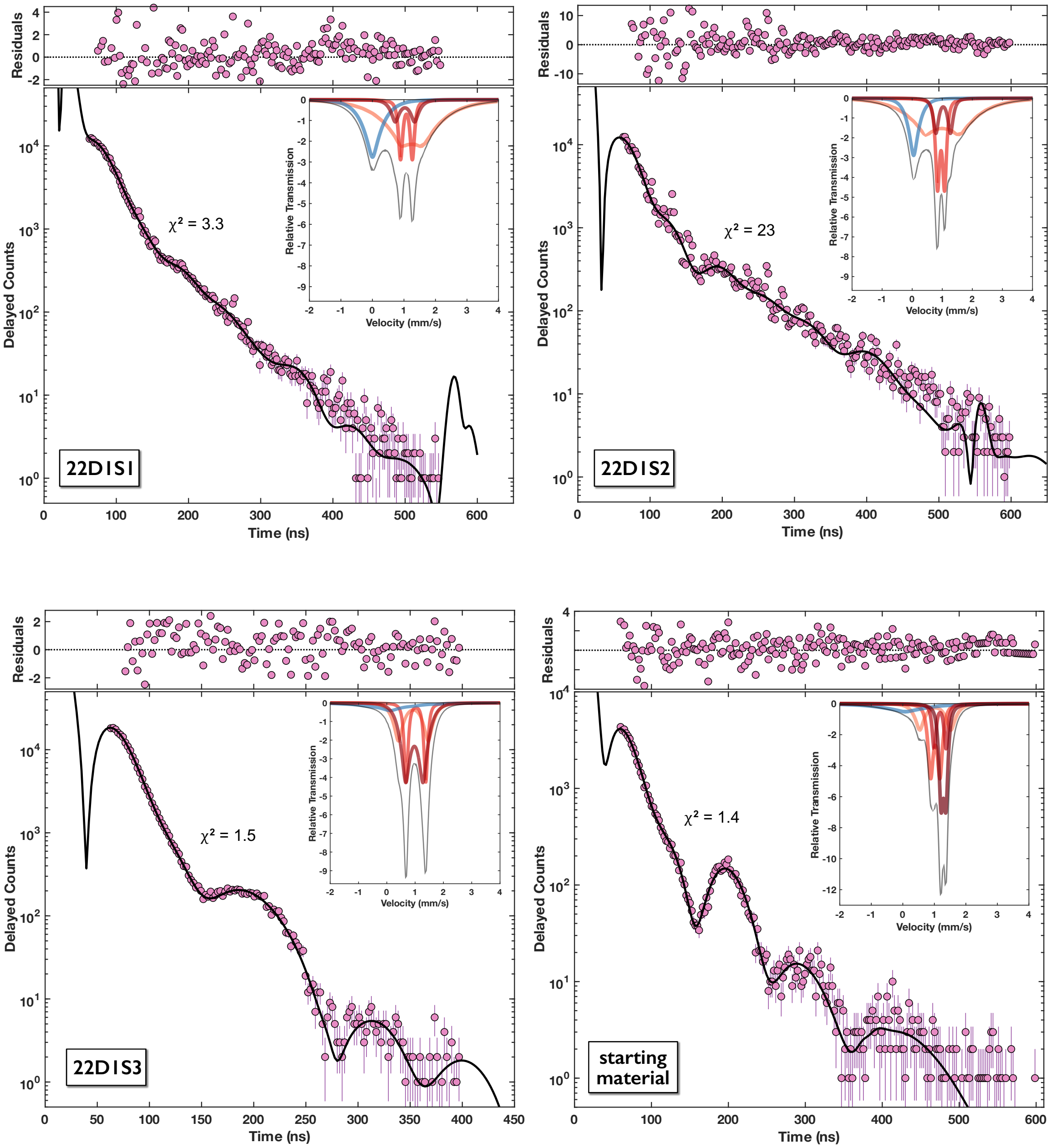

Fig. S12.
SMS time spectra (hybrid mode) 22D1S1 (top left), 22D1S2 (top right), 22D1S3 (bottom left), and starting material (bottom right) showing data (colored points) and best-fitting models (black lines). Insets show best-fitting models calculated in energy domain, showing the ferric iron lines in light blue. See Tables S11-14 for full details.

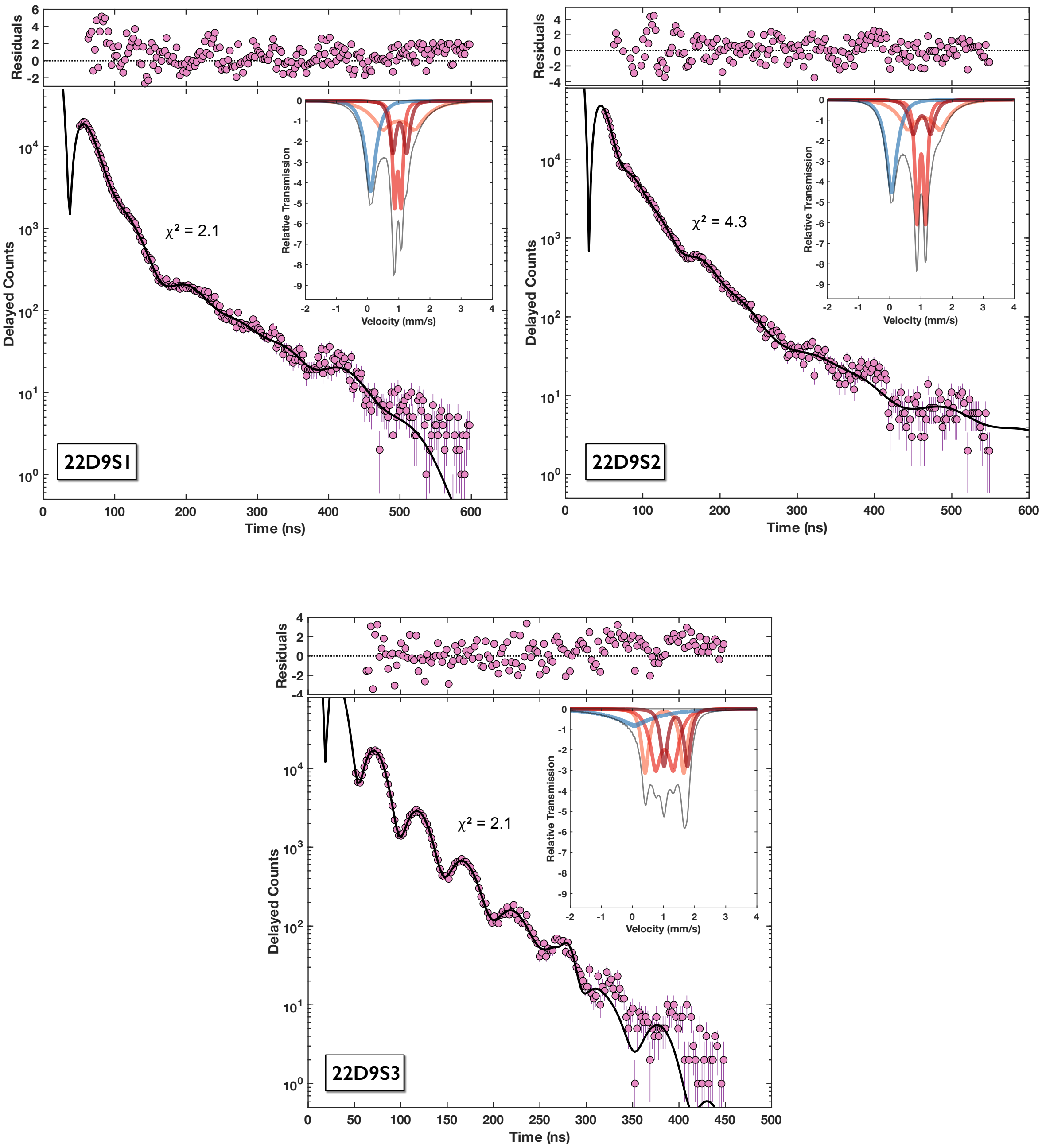

Fig. S13.
SMS time spectra (hybrid mode) 22D9S1 (top left), 22D9S2 (top right), and 22D9S3 (bottom), showing data (colored points) and best-fitting models (black lines). Insets show best-fitting models calculated in energy domain, showing the ferric iron lines in light blue. See Tables S8-10 for full details.

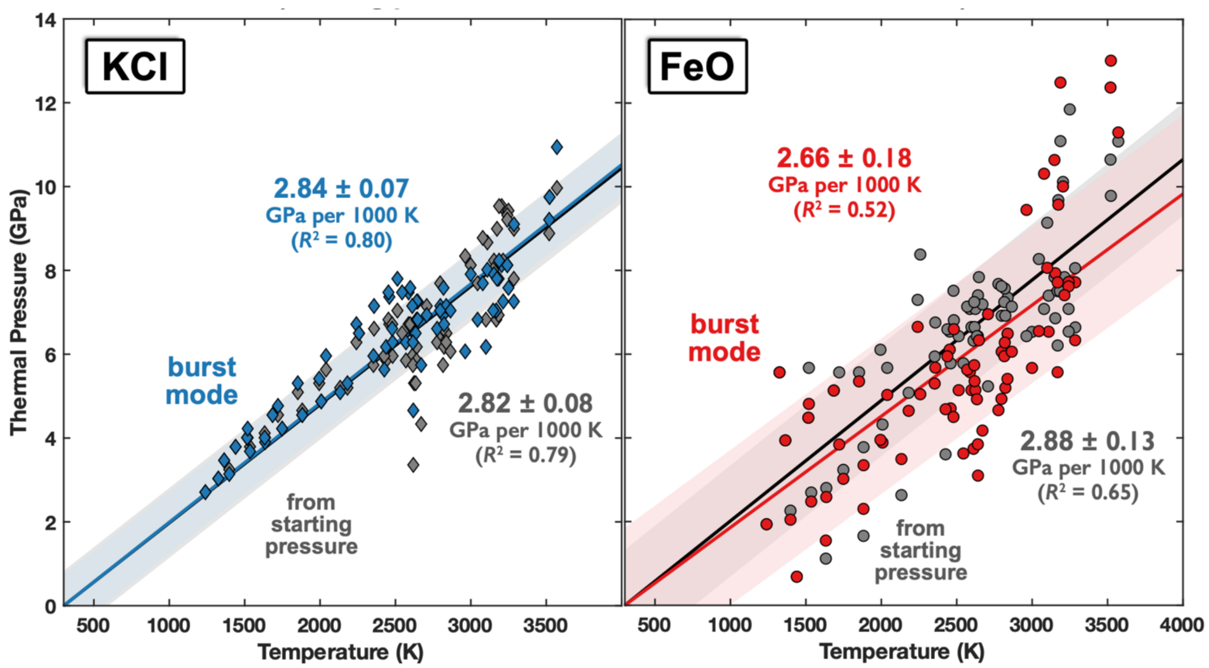

Fig. S14.

Thermal pressures from four representative XRD heating runs (starting pressures 32 GPa, 59 GPa, 66 GPa, 86 GPa). See Methods for details.

Table S1.
P-*T* conditions of the defect order-disorder transition in XRD heating runs.

**Heating run**	$P_{1,\,\mathrm{KCl}}$ **(GPa)**	$P_{1,\,\mathrm{FeO}}$ **(GPa)**	P_1 **(GPa)**	σP_1 **(GPa)**	T_1 **(K)**	σT_1 **(K)**
22D9S1	39.5	38.8	40	2	2570	130
22D9S2	37.7	37.4	38	1	2680	80
21D1S3	63.1	63.7	63	2	2950	250
21D1S2	64.5	65.1	65	2	3020	120
21D1S1	65.6	66.6	66	3	2950	150
22D1S2	68.5	68.3	68	2	3000	160
22D1S1	65.3	68.1	67	3	3050	150
20D4S2	72.9	69.9	71	4	3050	150
20D4S1	74.7	73.4	74	2	3150	150
20D4S3	74.7	74.4	75	1	3180	160
20D4S4	74.8	74.7	75	1	3200	200
21D7S1	91.9	95.0	94	5	3250	200
21D7S2	95.0	92.9	94	5	3250	160
21D8S1	113.4	113.6	114	4	3450	250
21D8S2	113.2	116.3	115	6	3480	220
21D8S3	112.8	120.6	117	8	3400	200
21D8S4	115.1	117.2	116	5	3500	200
22D7S1	131.7	134.2	133	6	3500	250
22D7S2	136.6	136.5	137	5	3550	220

Table S2.
P-T conditions of the melting transition in XRD heating runs.

Heating run	$P_{2,\,KCl}$ (GPa)	$P_{2,\,FeO}$ (GPa)	P_2 (GPa)	σP_2 (GPa)	T_2 (K)	σT_2 (K)
22D9S1	38.3	38.3	38	2	2800	120
22D9S2	34.0	38.1	36	4	2850	100
22D1S1	67.1	71.6	69	4	3500	150
20D4S3	75.8	76.8	76	3	3500	150
20D4S4	75.6	76.7	76	3	3550	150
21D8S1	115.7	119.5	118	4	3950	120
21D8S2	114.9	120.3	118	6	4000	200
21D8S4	116.1	120.8	118	5	3950	200
22D7S1	135.2	137.9	137	5	4050	200
22D7S2	138.8	140.1	140	6	4200	220

Table S3.
Best-fit results obtained from the synchrotron Mössbauer spectroscopy heating runs.

Heating run	Starting P_{ruby} (GPa)	P_{melt} (GPa)	σP_{melt} (GPa)	T_{melt} (K)	σT_{melt} (K)	χ^2
18D4P2	30	37	3	2920	105	0.37
18D4P3	40	48	3	3130	110	0.76
18D6S1	50	59	3	3400	110	1.64

Table S4.
Best-fit results for SMS time spectrum D4P2 (T = 1700 K, $f_{LM} = 0.33$) collected before heating run 18D4P2. Pressure is determined from ruby fluorescence measurements before and after heating and the thermal pressure model reported in this study.

P = 33 ± 2 GPa	Site 1 (ferrous)	Site 2 (ferrous)	Site 3 (ferric)	Site 4 (magnetic)
Weight	55 ± 3%	11%[a]	11 ± 4%	23 ± 5%
Weight (renormalized)[b]	72%	14%	14%	0%
Relative Isomer Shift (mm/s)	0	0	-0.98 ± 0.02	-0.98 ± 0.02[c]
Quadrupole Splitting (mm/s)	0.25[a]	1.18 ± 0.04	0	0
FWHM	0.03[a] mm/s	0.27 ± 0.03 mm/s	0.38 ± 0.02 mm/s	18 ± 2 T
Magnetic Hyperfine Field (T)	0	0	0	31.4 ± 0.5
Thickness (μm)	7.7 ± 0.4			
Reduced χ^2	1.8 ± 0.1			

[a]These values were fixed during fitting and optimized by iterative manual variation.
[b]Weights are renormalized to report relative contribution to the summed total of sites 1, 2, and 3.
[c]This value was fit using the same fit parameter as for the isomer shift of site 3.

Table S5.
Best-fit results for SMS time spectrum D4P3 (T = 1600 K, $f_{LM} = 0.40$) collected before heating run 18D4P3. Pressure is determined from ruby fluorescence measurements before and after heating and the thermal pressure model reported in this study.

P = 43 ± 2 GPa	Site 1 (ferrous)	Site 2 (ferrous)	Site 3 (ferric)	Site 4 (magnetic)
Weight	60 ± 4%	6.5%[a]	6.5 ± 3%	27 ± 2%
Weight (renormalized)[b]	82%	9%	9%	0%
Relative Isomer Shift (mm/s)	0	0	-0.81 ± 0.01	-0.81 ± 0.01[c]
Quadrupole Splitting (mm/s)	0.25[a]	1.21 ± 0.05	0	0
FWHM	0.03[a] mm/s	0.1[a] mm/s	0.1[a] mm/s	10.8 ± 0.4 T
Magnetic Hyperfine Field (T)	0	0	0	40.8 ± 0.2
Thickness (μm)	5.58 ± 0.09			
Reduced χ^2	1.9 ± 0.1			

[a]These values were fixed during fitting and optimized by iterative manual variation.
[b]Weights are renormalized to report relative contribution to the summed total of sites 1, 2, and 3.
[c]This value was fit using the same fit parameter as for the isomer shift of site 3.

Table S6.
Best-fit results for SMS time spectrum D6S1_1 (T = 1500 K, $f_{LM} = 0.47$) collected before heating run 18D6S1. Pressure is determined from ruby fluorescence measurements before and after heating and the thermal pressure model reported in this study.

P = 53 ± 2 GPa	Site 1 (ferrous)	Site 2 (ferrous)	Site 3 (ferric)	Site 4 (magnetic)
Weight	57 ± 6%	9%[a]	9 ± 4%	25 ± 2%
Weight (renormalized)[b]	76%	12%	12%	0%
Relative Isomer Shift (mm/s)	0	0	-0.80 ± 0.01	-0.80 ± 0.01[c]
Quadrupole Splitting (mm/s)	0.27 ± 0.04	1.26 ± 0.06	0	0
FWHM	0.05[a] mm/s	0.61 ± 0.06 mm/s	0.43 ± 0.05 mm/s	7.2 ± 0.2 T
Magnetic Hyperfine Field (T)	0	0	0	43.2 ± 0.1
Thickness (μm)	5.1 ± 0.3			
Reduced χ^2	3.2 ± 0.1			

[a]These values were fixed during fitting and optimized by iterative manual variation.
[b]Weights are renormalized to report relative contribution to the summed total of sites 1, 2, and 3.
[c]This value was fit using the same fit parameter as for the isomer shift of site 3.

Table S7.
Best-fit results for SMS time spectrum D6S1_2 (T = 1500 K, $f_{LM} = 0.47$) collected after heating run 18D6S1. Pressure is determined from ruby fluorescence measurements before and after heating and the thermal pressure model reported in this study.

P = 53 ± 2 GPa	Site 1 (ferrous)	Site 2 (ferrous)	Site 3 (ferric)	Site 4 (magnetic)
Weight	50 ± 6%	15%[a]	15 ± 4%	20 ± 2%
Weight (renormalized)[b]	62%	19%	19%	0%
Relative Isomer Shift (mm/s)	0	0	-0.81 ± 0.01	-0.81 ± 0.01[c]
Quadrupole Splitting (mm/s)	0.25[a]	0.78 ± 0.01	0	0
FWHM	0.05[a] mm/s	0.05[a] mm/s	0.45 ± 0.03 mm/s	6.6 ± 0.3 T
Magnetic Hyperfine Field (T)	0	0	0	41.1 ± 0.1
Thickness (μm)	3.3 ± 0.2			
Reduced χ^2	2.8 ± 0.1			

[a]These values were fixed during fitting and optimized by iterative manual variation.
[b]Weights are renormalized to report relative contribution to the summed total of sites 1, 2, and 3.
[c]This value was fit using the same fit parameter as for the isomer shift of site 3.

Table S8.
Best-fit results for SMS time spectrum 22D9S1 (P = 2.5, T = 300 K, $f_{LM} = 0.70$, melted at 35 GPa). Pressure is determined from ruby fluorescence measurements.

	Site 1 (ferrous)	Site 2 (ferrous)	Site 3 (ferric)	Site 4 (ferrous)
Weight (%)	31 ± 4	24	26 ± 1	19 ± 1
Relative Isomer Shift (mm/s)	0.97 ± 0.01	0.94 ± 0.01	0.09 ± 0.01	1[a]
Quadrupole Splitting (mm/s)	1.00 ± 0.02	0.20 ± 0.01	0[a]	0.43 ± 0.01
FWHM (mm/s)	0.58 ± 0.04	0.05[a]	0.24 ± 0.01	0.11 ± 0.01
Thickness (μm)	3.82 ± 0.09			
Reduced χ^2	2.1 ± 0.1			

[a]These values were fixed during fitting.

Table S9.
Best-fit results for SMS time spectrum 22D9S2 (P = 2.5, T = 300 K, $f_{LM} = 0.70$, melted at 35 GPa). Pressure is determined from ruby fluorescence measurements.

	Site 1 (ferrous)	Site 2 (ferrous)	Site 3 (ferric)	Site 4 (ferrous)
Weight (%)	28 ± 3	29	27 ± 2	16 ± 2
Relative Isomer Shift (mm/s)	1.06 ± 0.01	0.98 ± 0.01	0.06 ± 0.01	1[a]
Quadrupole Splitting (mm/s)	0.99 ± 0.02	0.267 ± 0.003	0[a]	0.18 ± 0.02
FWHM (mm/s)	0.48 ± 0.03	0.05[a]	0.24 ± 0.01	0.18 ± 0.02
Thickness (μm)	5.17 ± 0.09			
Reduced χ^2	4.3 ± 0.2			

[a]These values were fixed during fitting.

Table S10.
Best-fit results for SMS time spectrum 22D9S3 (P = 2.5, T = 300 K, $f_{LM} = 0.70$, unheated at 30 GPa). Pressure is determined from ruby fluorescence measurements.

	Site 1 (ferrous)	Site 2 (ferrous)	Site 3 (ferric)	Site 4 (ferrous)
Weight (%)	26 ± 2	35	18 ± 1	21 ± 2
Relative Isomer Shift (mm/s)	1[a]	1[a]	0.07 ± 0.03	1.35 ± 0.01
Quadrupole Splitting (mm/s)	1.2 ± 0.02	0.55 ± 0.01	0[a]	0.73 ± 0.02
FWHM (mm/s)	0.14 ± 0.01	0.26 ± 0.01	1.16 ± 0.01	0.12 ± 0.01
Thickness (μm)	11.6 ± 0.07			
Reduced χ^2	2.13 ± 0.1			

[a]These values were fixed during fitting.

Table S11.
Best-fit results for SMS time spectrum 22D1S1 (P = 2.8, T = 300 K, $f_{LM} = 0.70$, melted at 68 GPa). Pressure is determined from ruby fluorescence measurements.

	Site 1 (ferrous)	Site 2 (ferrous)	Site 3 (ferric)	Site 4 (ferrous)
Weight (%)	53 ± 3	14	24 ± 1	9 ± 1
Relative Isomer Shift (mm/s)	1.22 ± 0.01	1.05 ± 0.01	0.00 ± 0.01	1[a]
Quadrupole Splitting (mm/s)	0.67 ± 0.01	0.36 ± 0.01	0[a]	0.60 ± 0.01
FWHM (mm/s)	0.91 ± 0.01	0.05[a]	0.40 ± 0.01	0.17 ± 0.01
Thickness (μm)	7.9 ± 0.2			
Reduced χ^2	3.3 ± 0.1			

[a]These values were fixed during fitting.

Table S12.
Best-fit results for SMS time spectrum 22D1S2 (P = 2.8, T = 300 K, $f_{LM} = 0.70$, heated at 68 GPa to XRD T_1 (subsolidus defect order-disorder transition)). Pressure is determined from ruby fluorescence measurements.

	Site 1 (ferrous)	Site 2 (ferrous)	Site 3 (ferric)	Site 4 (ferrous)
Weight (%)	52 ± 10	21	16 ± 3	11 ± 2
Relative Isomer Shift (mm/s)	0.98 ± 0.03	0.93 ± 0.01	0.05 ± 0.01	1[a]
Quadrupole Splitting (mm/s)	1.11 ± 0.01	0.23 ± 0.02	0[a]	0.47 ± 0.02
FWHM (mm/s)	0.92 ± 0.01	0.05[a]	0.22 ± 0.02	0.09 ± 0.02
Thickness (μm)	4.7 ± 0.4			
Reduced χ^2	22.7 ± 0.3			

[a]These values were fixed during fitting.

Table S13.
Best-fit results for SMS time spectrum 22D1S3 (P = 2.8, T = 300 K, $f_{LM} = 0.70$, unheated at 60 GPa). Pressure is determined from ruby fluorescence measurements.

	Site 1 (ferrous)	Site 2 (ferrous)	Site 3 (ferric)	Site 4 (ferrous)
Weight (%)	23 ± 6	21	9 ± 7	47 ± 20
Relative Isomer Shift (mm/s)	0.91 ± 0.01	1[a]	0.11 ± 0.07	0.94 ± 0.01
Quadrupole Splitting (mm/s)	1.02 ± 0.04	0.67 ± 0.05	0[a]	0.59 ± 0.03
FWHM (mm/s)	0.23 ± 0.01	0.005[a]	1.14 ± 0.02	0.23 ± 0.03
Thickness (μm)	4.0 ± 0.3			
Reduced χ^2	1.5 ± 0.1			

[a]These values were fixed during fitting.

Table S14.
Best-fit results for the starting sample material (P = 1 bar, T = 300 K, $f_{LM} = 0.70$). Pressure is determined from ruby fluorescence measurements.

	Site 1 (ferrous)	Site 2 (ferrous)	Site 3 (ferric)	Site 4 (ferrous)	Site 5 (ferrous)
Weight (%)	18 ± 1	28	11.2 ± 0.4	13 ± 1	30 ± 3
Relative Isomer Shift (mm/s)	1[a]	1[a]	0.07 ± 0.02	1.15 ± 0.01	1.25 ± 0.01
Quadrupole Splitting (mm/s)	0.96 ± 0.01	0.29 ± 0.01	0[a]	0.37 ± 0.02	0.15[a]
FWHM (mm/s)	0.19 ± 0.01	0.078 ± 0.004	1.05 ± 0.01	0.04 ± 0.01	0.05[a]
Thickness (μm)	5.95 ± 0.03				
Reduced χ^2	1.38 ± 0.08				

[a]These values were fixed during fitting.
